# Comparison of Substance Use Disorder Diagnosis Rates From Electronic Health Record Data With Substance Use Disorder Prevalence Rates Reported in Surveys Across Sociodemographic Groups in the Veterans Health Administration

**DOI:** 10.1001/jamanetworkopen.2022.19651

**Published:** 2022-06-30

**Authors:** Emily C. Williams, Olivia V. Fletcher, Madeline C. Frost, Alex H. S. Harris, Donna L. Washington, Katherine J. Hoggatt

**Affiliations:** 1 Health Services Research and Development Center of Innovation for Veteran-Centered and Value-Driven Care, Veterans Affairs Puget Sound Health Care System, Seattle, Washington; 2 Department of Health Systems and Population Health, University of Washington School of Public Health, Seattle; 3 Center for Innovation to Implementation, Veterans Affairs Palo Alto Health Care System, Menlo Park, California; 4 Department of Surgery, Stanford University School of Medicine, Palo Alto, California; 5 Veterans Affairs Health Services Research and Development Center for the Study of Healthcare Innovation, Implementation, and Policy, Veterans Affairs Greater Los Angeles Healthcare System, Los Angeles, California; 6 Division of General Internal Medicine and Health Services Research, Department of Medicine, UCLA Geffen School of Medicine, Los Angeles, California; 7 San Francisco Veterans Affairs Health Care System, San Francisco, California; 8 Department of Medicine, University of California, San Francisco

## Abstract

**Importance:**

Substance use disorders (SUDs) are major contributors to morbidity and mortality globally, but they are often underrecognized and underdiagnosed, particularly in some sociodemographic subgroups. Understanding the extent to which clinical diagnoses underestimate these conditions within subgroups is imperative to achieving equitable treatment, regardless of race, ethnicity, gender, or age, and to informing and improving performance monitoring.

**Objective:**

To compare clinically documented diagnosis rates of alcohol use disorder (AUD), drug use disorder (DUD), and total SUD (AUD and/or DUD) with the prevalence of these disorders as reported in surveys—based on structured, validated diagnostic assessments—across demographic subgroups.

**Design, Setting, and Participants:**

A telephone-based survey was conducted from January 8, 2018, to April 30, 2019, among 5995 Veterans Health Administration (VHA) outpatients who were randomly sampled from 30 VHA facilities and were 18 years of age or older, could complete the survey in English, and had a valid address and telephone number. Survey data were linked to electronic health record (EHR) data for all participants. Statistical analysis was performed between January 29, 2020, and April 20, 2021.

**Exposures:**

Demographic subgroups based on self-report: gender (male or female), age (18-34, 35-49, 50-64, 65-74, and ≥75 years), and race and ethnicity (Black non-Hispanic, Hispanic, multiracial, other [Asian or Asian-American, American Indian or Alaskan Native, Native Hawaiian or Pacific Islander, and any other race endorsed by the participant], and White non-Hispanic).

**Main Outcomes and Measures:**

Survey-based prevalence rates of AUD, DUD, and SUD were assessed using the Mini International Neuropsychiatric Interview, version 7.0, the only validated instrument available at study outset that measured *Diagnostic and Statistical Manual of Mental Disorders* (Fifth Edition) criteria for past 12-month diagnoses. Clinically documented diagnosis rates were measured using *International Statistical Classification of Diseases and Related Health Problems, Tenth Revision* diagnoses from VHA EHR data. Analyses compared survey-based prevalence rates of AUD, DUD, and SUD with diagnosis rates using sensitivity and specificity and difference-in-difference analysis. All analyses were weighted with survey weights to account for nonresponse.

**Results:**

Of 5995 participants, 4115 (68.6%) were White non-Hispanic, and 5429 (91.1%) were male; the mean (SD) age was 61.5 (15.3) years. The survey-based prevalence rates of AUD, DUD, and SUD were higher than the diagnosis rates among all patients (AUD, 608 [10.1%] vs 360 [6.0%]; DUD, 282 [4.7%] vs 275 [4.6%]; SUD, 768 [12.8%] vs 515 [8.6%]). Survey-based prevalence rates of AUD and SUD exceeded the diagnosis rates in every demographic subgroup. Gaps between diagnosis rates and survey-based prevalence rates for AUD and SUD were largest among patients aged 18 to 34 years (AUD diagnosis rate, 27 [6.9%; 95% CI, 4.8%-9.9%] vs AUD prevalence rate, 88 [22.4%; 95% CI, 17.3%-28.5%]; SUD diagnosis rate, 41 [10.5%; 95% CI, 8.1%-13.4%] vs SUD prevalence rate, 109 [27.7%; 95% CI, 22.6%-33.3%]) and Hispanic and Latinx patients (AUD diagnosis rate, 31 [7.6%; 95% CI, 5.3%-10.8%] vs AUD prevalence rate, 72 [17.7%; 95% CI, 14.0%-22.1%]; and SUD diagnosis rate, 48 [11.7%; 95% CI, 7.9%-16.9%] vs SUD prevalence rate, 88 [21.6%; 95% CI, 18.0%-25.8%]). For DUD, only patients aged 18 to 34 years had a true prevalence rate that significantly exceeded the diagnosis rate (diagnosis rate, 21 [5.4%; 95% CI, 3.7%-7.8%] vs prevalence rate, 40 [10.1%; 95% CI, 7.2%-14.0%]).

**Conclusions and Relevance:**

The results of this survey study suggest that existing diagnostic procedures and tools are insufficient to capture SUD prevalence rates, particularly among younger patients and Hispanic and Latinx patients. Clinics and health systems should implement standardized SUD assessments to ensure the provision of equitable care and the optimal identification of underlying conditions for performance monitoring.

## Key Points

**Question:**

Is there variation in electronic health record (EHR) diagnosis rates of substance use disorders (SUDs) across sociodemographic groups in a geographically diverse sample of veteran outpatients?

**Findings:**

In a telephone-based survey study linked to EHR data, diagnosis rates of alcohol use disorder (AUD), drug use disorder, and combined SUDs were compared with the prevalence rates of these disorders as reported in surveys. The survey-based prevalence rates of each disorder exceeded the diagnosis rates in every demographic subgroup, with varying disparity across groups; disparities in AUD and SUD diagnosis rates were largest among the youngest patients and Hispanic and Latinx patients.

**Meaning:**

This study suggests that existing diagnostic procedures and tools are insufficient to capture the prevalence of SUD, particularly for young patients and Hispanic and Latinx patients, who may experience the greatest consequences of SUD.

## Teaser

This survey study compares clinically documented diagnosis rates of alcohol use disorder, drug use disorder, and total substance use disorder from electronic health record data with the prevalence rates of these disorders as reported in surveys, estimated using structured, validated diagnostic assessments across demographic subgroups.

## Introduction

Substance use disorders (SUDs) are harmful and stigmatized medical conditions.^1,2,3^ Both the prevalence and treatment of SUDs vary across subgroups. Although approximately 14.5 million US residents have alcohol use disorder (AUD), its prevalence is highest among young adults aged 18 to 25 years, American Indian and Alaska Native individuals, non-Hispanic White individuals, and men.^2,4^ Similarly, 8.3 million US residents have drug use disorders (DUDs), with higher prevalence among young adults aged 18 to 25 years, men, and some groups based on race and/or ethnic identity. Although effective SUD treatments can be delivered via primary and specialty care,^5,6,7^ SUDs have one of the largest treatment gaps for any medical condition,^8^ and given similar conditions, young adults, patients with minoritized racial and ethnic identities, and women may be less likely to receive treatments than their respective older, White, and male counterparts.^9,10,11,12,13^

Ensuring the equitable identification and diagnosis of any SUD in clinical settings can help address treatment inequity. Because measures of SUD treatment quality often rely on denominators composed of patients with clinically documented diagnoses, obtaining accurate estimates of individuals with SUD and understanding whether clinical diagnoses underestimate individuals with SUD across subgroups are imperative for monitoring equity in the provision of treatment. In the US, understanding patterns of SUD diagnosis across racial and ethnic groups is particularly important given the prominence of structural racism—historically rooted and culturally reinforced societal fostering of racism and discrimination through inequitable systems (eg, criminal justice)—in responses to substance use.^14,15^ Although one study has qualitatively compared the prevalence of clinically recognized AUD with national survey-based estimates across racial and ethnic groups,^12^ data have not been available to directly compare prevalence across sociodemographic subgroups within the same sample, to our knowledge.

The US Veterans Health Administration (VHA) is the largest integrated health care system^16^ offering a critical population for examining SUD diagnoses because SUD is common among veterans,^17^ improving SUD care is a priority, and the VHA—like other systems—uses care quality metrics that rely on clinically documented SUD diagnoses for quality improvement.^18^ In a large, diverse sample of VHA patients, we compared clinically documented diagnosis rates of AUD, DUD, and total SUD (AUD and/or DUD) with the prevalence rates estimated by a structured, validated diagnostic assessment across demographic subgroups.

## Methods

### Data Sources, Setting, and Sample

Patients from 30 VHA health care facilities were randomly sampled and recruited from January 8, 2018, to April 30, 2019, for a telephone survey administered by trained lay interviewers with prior experience surveying veterans about substance use. Facilities were purposively selected to represent US regions and included facilities with a range of SUD diagnosis rates and patient identity characteristics. Patients with a documented outpatient encounter at one of the facilities in the year prior to data extraction were eligible if they were 18 years of age or older and able to complete the survey in English and provide a valid address and telephone number. All eligible veterans were required to provide informed consent orally via telephone to participate. Additional diagnostic electronic health record data for up to 1 year prior to the survey were extracted for all patients who provided oral consent via telephone and completed the survey. This study followed the American Association for Public Opinion Research (AAPOR) reporting guideline, incorporated AAPOR standards,^19^ used AAPOR formulas to compute target response rates for telephone surveys, was approved by VHA institutional review boards at greater Los Angeles, Puget Sound, and San Francisco facilities, and was peer reviewed and funded by the VHA Health Services Research and Development Service.^20^

### Measures

#### Survey-Based Prevalence

Surveyed patients completed the Mini International Neuropsychiatric Interview, version 7.0 (MINI 7.0),^21^ which assessed *Diagnostic and Statistical Manual of Mental Disorders* (Fifth Edition) (*DSM-5*) criteria for AUD and substance-specific DUD during the 12 months prior to the survey. The MINI 7.0 was chosen instead of 3 other instruments that measured *DSM* criteria (the World Health Organization’s Composite International Diagnostic Interview [CIDI], the Structured Clinical Interview for *DSM-IV*–Research Version [SCID-RV], and the Alcohol Use Disorder and Associated Disabilities Interview Schedule), based on its strong performance relative to both the SCID-RV and the CIDI (eg, sensitivity and specificity range of 0.83-0.97 for current AUD), the lack of *DSM-5* versions of the CIDI and SCID-RV (for research) when the study was fielded, and its relative decreased participant burden.^21,22,23,24^ Per protocol, the MINI 7.0 assessed AUD among patients reporting consuming 3 or more alcoholic drinks within 3 hours on 3 or more occasions in the past 12 months. Patients reporting any lifetime use of a nonalcohol, nontobacco drug and use of that drug more than once in the past 12 months to get high, get a buzz, feel elated, or change a mood were assessed for DUD related to that drug. Patients were classified as having AUD or DUD if they scored 2 or more on the relevant MINI 7.0 section consistent with the *DSM-5*. Patients reporting using more than 1 drug were assessed for whichever drug was causing the biggest problems or being used the most. Patients who met criteria for any SUD (AUD, DUD, or both) were classified as having SUD.

#### Clinically Documented Diagnosis Rates

Electronic health record data were used to identify clinically documented diagnosis rates of AUD, DUD, and any SUD using *International Statistical Classification of Diseases and Related Health Problems, Tenth Revision* (*ICD-10*) codes documented on the day of or in the year prior to the date of the survey for each participant. Alcohol use disorder was identified with *ICD-10* codes F10.X and DUD was identified with codes F11.X to F16.X and codes F18.X to F19.X (excluding nicotine dependence), whereas any SUD was defined as the presence of a code for AUD or DUD. The *ICD-10* codes included those for substance use as well as those for remission conditions.

#### Sociodemographic Groups

Sociodemographic characteristics were self-reported on survey measures. Gender was categorized as male or female (although 4 respondents self-identified as transgender, they were excluded owing to the small number). Age groups were generated consistent with prior national surveys,^2,25^ adding a category for the oldest veterans (18-34, 35-49, 50-64, 65-74, and ≥75 years). Participants were asked about both ethnicity (Hispanic vs non-Hispanic) and race (American Indian or Alaska Native, Asian, Black, multiracial, Native Hawaiian or Other Pacific Islander, respondent-specified race, and White). Owing to very low numbers of patients identifying with some racial groups, and consistent with census recommendations regarding prioritization of ethnicity and race, we combined race and ethnicity into the following categories: Black non-Hispanic, Hispanic, multiracial, other (Asian or Asian-American, American Indian or Alaskan Native, Native Hawaiian or Pacific Islander, and any other race endorsed by the participant), and White non-Hispanic.

### Statistical Analysis

Statistical analysis was performed from January 29, 2020, to April 20, 2021. All analyses incorporated person-level weights to account for sampling design and survey nonresponse. Descriptive statistics described sample characteristics. In primary analyses, we computed point estimates and 95% CIs for both survey-based prevalence and clinically documented diagnosis rates for AUD, DUD, and SUD in each subgroup. To assess performance of clinically documented diagnoses, we also calculated point estimates and 95% CIs of the sensitivity and specificity of clinically documented diagnoses (relative to survey-based prevalence as the referent standard) for each subgroup. We compared sensitivity and specificity across categories of each sociodemographic variable using joint Wald tests. In supplemental analyses assessing disparities in recognition of SUDs, we computed point and 95% CI estimates of differences between diagnosis rates and prevalence rates for each subgroup and assessed statistical differences in the differences between subgroups (eg, whether the gap between clinical and survey-based diagnoses differed for patients reporting Black race relative to those reporting White race) using nonparametric bootstrapping with repeated sampling (1000 replicates) to compare each subgroup with the reference group (male [gender], aged 18-34 years [age], and non-Hispanic White [race and ethnicity]). All statistical tests were 2-sided with an α of .05. Analyses were conducted using Stata/SE, version 15 (StataCorp LLC).

## Results

Among 5995 of 6000 participants (99.9%) with complete data (response rate, 51%; range, 44%-56% across subgroups), 4115 (68.6%) were White non-Hispanic and 5429 (91.1%) were male; the mean (SD) age was 61.5 (15.3) years (Table 1).^26^ The diagnosis rate was 6.0% (n = 360) for AUD, 4.5% (n = 275) for DUD, and 8.5% (n = 515) for SUD; the survey-based prevalence rate was 10.1% (n = 608) for AUD, 4.7% (n = 282) for DUD, and 12.8% (n = 768) for SUD. Survey-based prevalence rates exceeded diagnosis rates for nearly all subgroups (Table 2; eTable 1 in the Supplement).

**Table 1.** Demographic Characteristics and Prevalence and Diagnosis Rates of AUD, DUD, and SUD in a Survey-Respondent Veterans Health Administration Outpatient Population

Characteristic	No. (%) (N = 5995)^a^
Gender^b^	
Male	5429 (91.1)
Female	566 (8.8)
Age (continuous), mean (SD), y	61.5 (15.3)
Age, y	
18-34	393 (6.7)
35-49	855 (13.8)
50-64	1517 (24.7)
65-74	2036 (34.4)
≥75	1194 (20.4)
Race and ethnicity	
Black non-Hispanic	1064 (17.3)
Hispanic or Latinx	410 (6.9)
Multiracial	241 (4.0)
White non-Hispanic	4115 (68.6)
Other^c^	165 (3.2)
Prevalence rate based on referent-standard survey	
AUD	608 (10.1)
DUD^d^	282 (4.7)
SUD^e^	768 (12.8)
Clinically documented diagnosis rate	
AUD	360 (6.0)
DUD^d^	275 (4.5)
SUD^e^	515 (8.5)
Lifetime substance use (N = 6000)^f^	
Alcohol	5467 (91.1)
Cannabis	2526 (41.9)
Cocaine	989 (16.4)
Hallucinogens	764 (12.7)
Stimulants	697 (11.6)
Opiates	499 (8.3)
Sedatives	440 (7.3)
Miscellaneous drugs	341 (5.7)
Inhalants	200 (3.3)
Dissociative drugs	186 (3.1)

Abbreviations: AUD, alcohol use disorder; DUD, drug use disorder; SUD, substance use disorder.

^a^ All analyses were weighted with survey weights to account for nonresponse; thus, the percentages reported do not necessarily equal number/total number.

^b^ Transgender persons have been excluded for analyses owing to small sample size (n = 4), and 1 person with missing gender was excluded from the analysis.

^c^ Includes Asian or Asian American, American Indian or Alaskan Native, Native Hawaiian or Pacific Islander, and any other race endorsed by participant.

^d^ Includes use of drugs in the following classes: cannabis, cocaine, stimulants, inhalants, tranquilizers, hallucinogens, opiates and narcotics, dissociative drugs, and other miscellaneous substances of abuse, excluding tobacco.

^e^ Includes AUD and DUD combined.

^f^ Statistics previously reported.^26^

**Table 2.** Clinically Documented Diagnosis Rates and Survey-Based Prevalence Rates of AUD, DUD, and SUD and Performance of Clinically Documented Diagnoses in a Survey-Respondent Veterans Health Administration Outpatient Population from 30 Geographically Diverse Facilities

Characteristic	No. (%) [95% CI]		Sensitivity, % (95% CI)	*P* value, absolute difference sensitivity	Specificity, % (95% CI)	*P* value, absolute difference specificity
	Clinically documented diagnoses	Survey-based prevalence (referent standard)				
**AUD**						
Overall	360 (6.0) [5.3-6.7]	608 (10.1) [9.2-11.1]	30.9 (27.3-34.8)	NA	96.9 (96.3-97.4)	NA
Gender						
Male	346 (6.3) [5.5-7.2]	568 (10.4) [9.5-11.4]	31.5 (27.6-35.6)	.14	96.6 (96.0-97.2)	<.001
Female	14 (2.5) [1.6-3.9]	40 (7.0) [5.3-9.1]	22.4 (13.1-35.7)		99.0 (97.6-99.6)	
Age, y						
18-34	27 (6.9) [4.8-9.9]	88 (22.4) [17.3-28.5]	23.7 (17.0-32.1)	.15	97.9 (95.2-99.1)	<.001
35-49	86 (10.1) [8.6-11.8]	153 (18.1) [15.6-20.9]	31.8 (24.9-39.5)		94.7 (93.0-96.0)	
50-64	123 (8.1) [6.4-10.2]	170 (11.2) [9.6-13.1]	33.4 (26.0-41.6)		95.1 (93.0-96.6)	
65-74	104 (5.1) [4.1-6.4]	161 (7.9) [6.6-9.5]	33.5 (26.0-41.9)		97.3 (96.6-97.9)	
≥75	20 (1.7) [1.1-2.6]	36 (3.0) [2.2-4.1]	22.5 (11.8-38.5)		98.9 (98.3-99.4)	
Race and ethnicity						
Black non-Hispanic	95 (9.0) [7.7-10.4]	139 (13.0) [10.9-15.6]	36.1 (31.2-41.4)	.02	95.1 (93.4-96.4)	.09
Hispanic and Latinx	31 (7.6) [5.3-10.8]	72 (17.7) [14.0-22.1]	26.1 (17.8-36.6)		96.4 (93.0-98.1)	
White non-Hispanic	206 (5.0) [4.1-5.9]	353 (8.6) [7.4-9.8]	30.2 (25.7-35.1)		97.4 (96.8-97.9)	
Multiracial	21 (8.8) [5.7-13.4]	23 (9.6) [6.6-13.8]	44.4 (25.5-65.1)		95.0 (91.2-97.1)	
Other	7 (4.0) [2.2-7.4]	21 (11.9) [8.7-16.2]	12.6 (5.0-28.4)		97.1 (93.6-98.7)	
**DUD**						
Overall	275 (4.5) [3.7-5.6]	282 (4.7) [3.9-5.6]	35.6 (27.2-45.0)		97.0 (96.3-97.5)	
Gender						
Male	259 (4.7) [3.8-5.7]	264 (4.8) [4.0-5.8]	35.7 (27.1-45.4)	.87	96.9 (96.2-97.4)	.03
Female	16 (2.9) [1.6-5.3]	18 (3.3) [2.1-5.1]	34.0 (16.7-57.0)		98.2 (96.4-99.1)	
Age, y						
18-34	21 (5.4) [3.7-7.8]	40 (10.1) [7.2-14.0]	20.4 (9.3-39.1)	.07	96.3 (93.7-97.7)	<.001
35-49	53 (6.2) [5.0-7.7]	64 (7.6) [5.8-9.8]	41.7 (29.2-55.4)		96.7 (95.6-97.6)	
50-64	109 (7.2) [5.4-9.4]	102 (6.7) [5.2-8.7]	38.1 (27.2-50.4)		95.1 (93.4-96.3)	
65-74	81 (4.0) [2.9-5.5]	72 (3.6) [2.7-4.6]	34.6 (23.0-48.5)		97.2 (95.7-98.2)	
≥75	11 (0.9) [0.5-1.7]	4 (0.3) [0.1-1.1]	50.2 (18.8-81.5)		99.3 (98.6-99.6)	
Race and ethnicity						
Black non-Hispanic	79 (7.5) [6.0-9.3]	79 (7.4) [5.6-9.8]	43.1 (33.7-53.0)	.01	95.4 (94.0-96.5)	.06
Hispanic and Latinx	28 (6.8) [3.3-13.5]	33 (8.1) [6.0-10.8]	32.8 (15.4-56.9)		95.5 (90.6-97.9)	
White non-Hispanic	139 (3.3) [2.8-4.0]	136 (3.3) [2.7-4.1]	28.4 (21.2-36.7)		97.5 (96.8-98.1)	
Multiracial	18 (7.5) [5.1-10.9]	21 (8.7) [5.7-13.1]	48.5 (27.9-69.6)		96.4 (93.6-98.0)	
Other	11 (6.0) [3.1-11.4]	13 (6.9) [3.9-11.7]	53.8 (25.2-80.1)		97.5 (91.6-99.3)	
**SUD**						
Overall	515 (8.5) [7.5-9.6]	768 (12.8) [11.7-13.9]	34.4 (30.8-38.1)		95.3 (94.5-96.0)	
Gender						
Male	490 (8.9) [7.9-10.1]	716 (13.1) [12.0-14.3]	35.0 (31.3-38.9)	.06	95.0 (94.2-95.8)	.001
Female	25 (4.5) [3.0-6.6]	52 (9.2) [7.2-11.8]	25.2 (17.0-35.9)		97.7 (95.8-98.7)	
Age, y						
18-34	41 (10.5) [8.1-13.4]	109 (27.7) [22.6-33.3]	23.9 (16.8-32.9)	.02	94.7 (91.3-96.8)	<.001
35-49	110 (12.9) [11.2-14.8]	187 (22.1) [19.6-24.8]	36.6 (30.0-43.8)		93.8 (92.0-95.3)	
50-64	180 (11.8) [9.7-14.3]	230 (15.2) [13.5-17.1]	37.3 (30.2-45.1)		92.7 (90.5-94.5)	
65-74	155 (7.6) [6.2-9.3]	203 (10.0) [8.5-11.7]	36.4 (29.7-43.6)		95.6 (94.2-96.7)	
≥75	29 (2.4) [1.7-3.4]	39 (3.2) [2.3-4.5]	25.9 (14.5-41.8)		98.3 (97.6-98.9)	
Race and ethnicity						
Black non-Hispanic	137 (13.0) [11.2-15.0]	182 (17.1) [14.8-19.6]	42.0 (35.5-48.7)	.05	93.0 (90.8-94.7)	.07
Hispanic and Latinx	48 (11.7) [7.9-16.9]	88 (21.6) [18.0-25.8]	29.2 (21.4-38.5)		93.2 (87.7-96.3)	
White non-Hispanic	288 (6.9) [6.1-7.9]	434 (10.5) [9.4-11.8]	31.4 (27.7-35.4)		96.0 (95.1-96.6)	
Multiracial	29 (12.2) [9.0-16.4]	36 (15.0) [11.3-19.7]	48.8 (33.1-64.7)		94.3 (90.1-96.7)	
Other	13 (7.3) [4.5-11.4]	28 (15.6) [11.5-20.9]	30.1 (18.2-45.4)		97.0 (93.2-98.7)	

Abbreviations: AUD, alcohol use disorder; DUD, drug use disorder; NA, not applicable; SUD, substance use disorder.

### Alcohol Use Disorder

Survey-based AUD prevalence rates generally exceeded diagnosis rates across demographic subgroups (Table 2; Figure). The largest difference between the diagnosis rate and the survey-based AUD prevalence rate was for patients aged 18 to 34 years, for whom the prevalence rate was more than 3 times as high as the diagnosis rate (88 [22.4%; 95% CI, 17.3%-28.5%] vs 27 [6.9%; 95% CI, 4.8%-9.9%]). In addition, the prevalence rate was more than 2 times as high as the diagnosis rate among the Hispanic and Latinx population (72 [17.7%; 95% CI, 14.0%-22.1%] vs 31 [7.6%; 95% CI, 5.3%-10.8%]), and approximately 3 times as high in those with other race and ethnicity (21 [11.9%; 95% CI, 8.7%-16.2%] vs 7 [4.0%; 95% CI, 2.2%-7.4%]).

**Figure.**
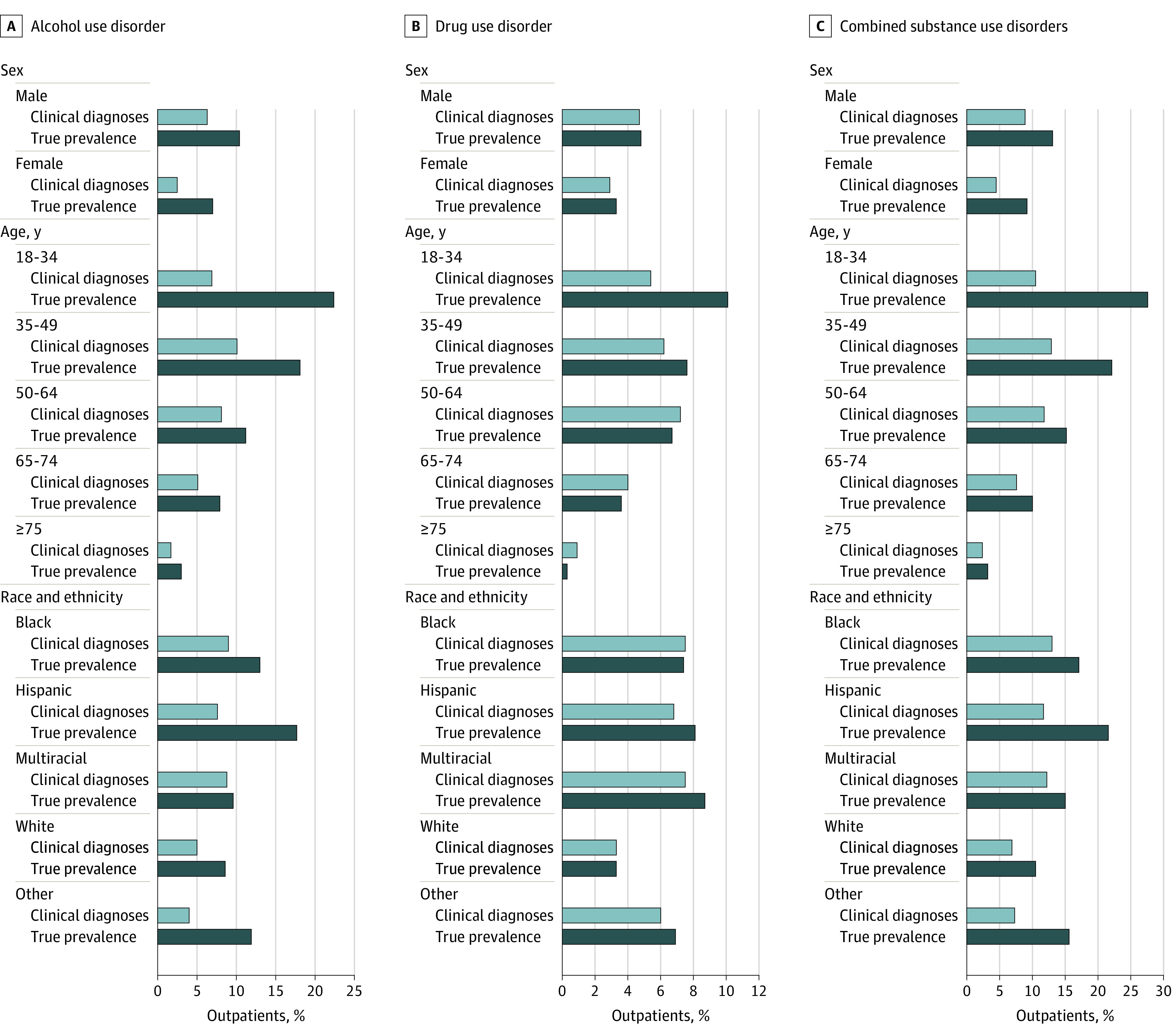
Difference Between Clinically Documented Diagnosis Rates and Survey-Based Prevalence Rates of Alcohol Use Disorder, Drug Use Disorder, and Combined Substance Use Disorders Across Demographic Subgroups in a Survey-Respondent Veterans Health Administration Outpatient Population From 30 Geographically Diverse Facilities

The sensitivity of diagnoses for identifying AUD was less than 45% for all subgroups (Table 2), with the lowest among women (22.4% [95% CI, 13.1%-35.7%]), patients aged 18 to 34 years (23.7% [95% CI, 17.0%-32.1%]), patients aged 75 years or older (22.5% [95% CI, 11.8%-38.5%]), and patients categorized as having other race and ethnicity (12.6% [95% CI, 5.0%-28.4%]). Relative to survey-based prevalence, the sensitivity of AUD diagnosis was highest among men (31.5% [95% CI, 27.6%-35.6%]), patients aged 50 to 64 years (33.4% [95% CI, 26.0%-41.6%]), patients aged 65 to 74 years (33.5% [95% CI, 26.0%-41.9%]), and multiracial patients (44.4% [95% CI, 25.5%-65.1%]). Specificity of AUD diagnosis was greater than 94% across all demographic subgroups.

Difference-in-difference analyses (eTable 1 in the Supplement) found little variability across gender but significant differences by age. The largest comparison was between the oldest (≥75 years) and youngest (18-34 years) age groups. For race and ethnicity, only Hispanic patients differed significantly from the referent group regarding diagnoses and prevalence.

### Drug Use Disorder

The survey-based prevalence rates of DUD exceeded the diagnosis rates across most subgroups; however, the prevalence rate was lower than the diagnosis rate for patients aged 50 to 64 years (102 [6.7%; 95% CI, 5.2%-8.7%] vs 109 [7.2%; 95% CI, 5.4%-9.4%]), patients aged 65 to 74 years (72 [3.6%; 95% CI, 2.7%-4.6%] vs 81 [4.0%; 95% CI, 2.9%-5.5%]), patients aged 75 years or older (4 [0.3%; 95% CI, 0.1%-1.1%] vs 11 [0.9%; 95% CI, 0.5%-1.7%]), and patients identifying as Black non-Hispanic (79 [7.4%; 95% CI, 5.6%-9.8%] vs 79 [7.5%; 95% CI, 6.0%-9.3%]) (Table 2). Among White non-Hispanic patients, the survey-based prevalence rate and the diagnosis rate were equal (136 [3.3%; 95% CI, 2.7%-4.1%] vs 139 [3.3%; 95% CI, 2.8%-4.0%]). The DUD prevalence rate was nearly twice as high as the diagnosis rate among patients aged 18 to 34 years (40 [10.1%; 95% CI, 7.2%-14.0%] vs 21 [5.4%; 95% CI, 3.7%-7.8%]), which also had the greatest difference between prevalence rate and diagnosis rate (the survey-based prevalence rate was 4.7 percentage points higher than the clinical diagnosis rate; *P* = .01). With the exception of the oldest age group, gaps between the diagnosis rate and the prevalence rate were low or nonexistent in other groups (<1.4 percentage points), with the smallest differences among men, Black non-Hispanic veterans, and White non-Hispanic veterans.

The sensitivity of clinical diagnoses was less than 54% for identifying underlying DUD among all subgroups, with the lowest being among women (34.0% [95% CI, 16.7%-57.0%]), patients aged 18 to 34 years (20.4% [95% CI, 9.3%-39.1%]), and White non-Hispanic patients (28.4% [95% CI, 21.2%-36.7%]). Sensitivity was highest among men (35.7% [95% CI, 27.1%-45.4%]), patients aged 75 years or older (50.2% [95% CI, 18.8%-81.5%]), and patients categorized as having other race or ethnicity (53.8% [95% CI, 25.2%-80.1%]). The specificity was greater than 95% for DUD across all subgroups. Difference-in-difference analyses identified little variability across gender and racial and ethnic groups (eTable 1 in the Supplement) but highlighted differences by age.

### Combined SUDs

The survey-based prevalence rates of SUD exceeded the clinical diagnosis rates across every subgroup (Figure; Table 2). For example, the prevalence rate exceeded the diagnosis rate for men (716 [13.1%; 95% CI, 12.0%-14.3%] vs 490 [8.9%; 95% CI, 7.9%-10.1%]), while for women, the prevalence rate was twice the diagnosis rate (52 [9.2%; 95% CI, 7.2%-11.8%] vs 25 [4.5%; 95% CI, 3.0%-6.6%]). The SUD prevalence rate was nearly twice as high as the diagnosis rate among those aged 35 to 49 years (187 [22.1%; 95% CI, 19.6%-24.8%] vs 110 [12.9%; 95% CI, 11.2%-14.8%]) and among Hispanic and Latinx persons (88 [21.6%; 95% CI, 18.0%-25.8%] vs 48 [11.7%; 95% CI, 7.9%-16.9%]) and was more than twice as high among patients aged 18 to 34 years (109 [27.7%; 95% CI, 22.6%-33.3%] vs 41 [10.5%; 95% CI, 8.1%-13.4%]) and among persons of other race and ethnicity (28 [15.6%; 95% CI, 11.5%-20.9%] vs 13 [7.3%; 95% CI, 4.5%-11.4%]).

The sensitivity of clinically documented diagnoses for identifying underlying SUD was less than 49% in all subgroups, with the lowest among women (25.2% [95% CI, 17.0%-35.0%]), patients aged 18 to 34 years (23.9% [95% CI, 16.8%-32.9%]), patients aged 75 years or older (25.9% [95% CI, 14.5%-41.8%]), and those identifying as Hispanic and Latinx (29.2% [95% CI, 21.4%-38.5%]) and other race and ethnicity (30.1% [95% CI, 18.2%-45.4%]). Sensitivity was highest among men (35.0% [95% CI, 31.3%-38.9%]), those aged 50 to 64 years (37.3% [95% CI, 30.2%-45.1%]), and patients identifying as multiracial (48.8% [95% CI, 33.1%-64.7%]). Specificity for SUD was greater than 92% across all subgroups.

We observed little difference in the gap between the SUD prevalence rate and the SUD diagnosis rate across gender, but age groups differed (eTable 1 in the Supplement). Among racial and ethnic groups, only Hispanic and Latinx patients had a statistically significant difference in the gap between the prevalence rate and the diagnosis rate relative to the reference group.

### Post Hoc Analyses

Because diagnosis may be challenging for clinicians around the diagnostic threshold, we performed post hoc analyses focusing only on patients with moderate or severe disorders identified by the survey (eTable 2 in the Supplement). The survey-based prevalence of SUD decreased owing to the increased threshold for diagnoses, and observed differences were somewhat attenuated. However, overall patterns remained, with similar differences between clinically documented diagnosis rates and survey-based prevalence rates among key subgroups (women, Hispanic and Latinx patients, and younger patients).

## Discussion

To our knowledge, this is the first study (and first data set in which it was possible) to investigate underdiagnosis of SUD across key sociodemographic subgroups by directly comparing self-reported structured diagnostic assessments with clinically documented diagnoses within the same sample. In this large diverse sample, clinically documented diagnosis rates for AUD, DUD, and combined SUDs were generally lower than the survey-based prevalence rates (reference standard), and clinical diagnoses had low sensitivity but high specificity for identifying the underlying substance use condition in nearly all subgroups. Differences between the diagnosis rate and the prevalence rate were larger in magnitude for some key subgroups for whom substance use may be particularly important to address, particularly younger and Hispanic and Latinx patients.

Although specificity of clinically documented diagnoses was high (>92%) for all conditions across all subgroups, sensitivity did not exceed 54% in any subgroup suggesting pervasive underdiagnosis across all groups and greater levels of underdiagnosis in some. The lowest sensitivities (most underdiagnosis) for AUD were observed among women, the youngest and oldest age groups, persons of other race and ethnicity, and White non-Hispanic persons. For DUD, the greatest levels of underdiagnosis were observed among the youngest age group and among persons reporting White non-Hispanic race and ethnicity. For SUD, the lowest estimates for sensitivities were for women, the youngest and oldest age groups, and those reporting Hispanic and Latinx ethnicity. These findings are concerning given that alcohol use is increasing most among women and young people,^27,28^ that the opioid epidemic and increased use of stimulants are more common (although associated with fewer consequences) among persons identifying as White relative to other groups, and that Hispanic persons are at greater risk for adverse substance-related consequences than non-Hispanic persons.^2,25,29^ Improvement in clinical recognition of these conditions (or better, improvement in recognition of use that increases risk for disorder) is needed, particularly for higher-risk subgroups.

Conversely, in this study, persons who reported being Black or multiple race and ethnicity, as well as men, had greater correspondence between reported prevalence and clinical diagnoses (eg, higher rates of clinical recognition) across all conditions, as did Hispanic and Latinx persons for DUD. Although these findings may reflect variations in practice across clinics where different subgroups receive care (eg, women and older veterans largely receive care in women-specific and geriatric clinics, respectively, and variations exist in the distribution of racial and ethnic groups across geographic regions in the VHA), findings may also reflect clinician-specific characteristics or practices, including differential documentation resulting from fear of harming or stigmatizing patients and/or internalized biases and/or direct discrimination or racism at play among clinicians during assessment for and diagnosis of SUD. Particularly regarding patients from minoritized racial and ethnic groups, structural racism has fundamentally shaped responses to substance use in the US through differential enforcement of drug policy (eg, disproportionate arrests or incarcerations and differential requirements for mandatory substance use treatment) and resulted in differential access to care for persons dependent on their race and/or ethnicity.^30^ Structural racism reinforces a cultural and interpersonal-level stigma regarding SUDs for some groups (eg, members of minoritized racial and ethnic groups) more than others.^15,31,32,33^ Although having fewer persons in minoritized racial and/or ethnic groups who were underdiagnosed may be associated with benefits via “higher-quality” care for SUDs—and it is important to recognize and treat SUDs for these patients (eg, drug overdose deaths are increasing more rapidly among Black people relative to most other racialized groups)^14,34^—such care may also reflect individual-level manifestations of stigma, discrimination, and racism in health care, which are associated with adverse outcomes,^35^ including increased severity of disease.^36^ Efforts are needed to ensure that clinical diagnoses of SUDs among persons in minoritized racial and/or ethnic groups result in improved SUD care and reduced adverse outcomes while not increasing harms.

Our findings have implications for clinical care and quality improvement. Clinicians should be aware that many patients—particularly Hispanic and Latinx patients and young patients—with SUD go unidentified, which can influence multiple other care-relevant health outcomes. Clinicians may also recognize SUDs more readily among patients whom they expect to have SUDs based on internalized biases and beliefs. Clinics and health systems should implement standard structured assessments for AUD and DUD to ensure the provision of equitable care and the optimal identification of underlying conditions. Similarly, performance monitoring relying on diagnosis-based measures to quantify patients eligible for care and inform and support quality improvement may be compromised, particularly for some groups. Results suggest that diagnosis-based measures are limited as an accurate reflection of SUD burden to inform delivery of SUD-related care, particularly among subgroups at greatest risk for adverse outcomes (eg, young persons at greatest risk of acute outcomes, such as injury). Other studies finding similar biases of diagnosis-based performance indicators have recommended population-based denominators for quality metrics.^37,38^

### Limitations

This study has several limitations. First, differences between survey-based and clinically documented diagnoses may be partially due to the diagnostic criteria used, given that *DSM-5* criteria may capture a higher prevalence than the *Diagnostic and Statistical Manual of Mental Disorders* (Fourth Edition) (*DSM-IV*),^39^ and our instrumentation did not include assessment of *DSM-IV*. Second, per MINI 7.0 protocol, survey assessment of AUD was limited to those reporting recent heavy drinking episodes, whereas for DUD, it was any drug use in the past year. Although it seems unlikely that someone with lower levels of consumption would meet AUD criteria, this assessment may have underestimated survey-based AUD. Third, owing to the slight lag time (approximately 2 months) between outpatient appointments indicating study eligibility and the survey, comparison measures may not reflect entirely overlapping time frames. Fourth, our sample was limited regarding nonbinary or transgender patients and some subgroups (eg, American Indian or Alaska Native persons as well as women). Especially given findings regarding greater underdiagnosis of AUD among patients of other race and ethnicity and differences identified by gender, further work should explore issues related to SUD underdiagnosis within more refined groups, some of whom (eg, women, transgender individuals, and American Indian or Alaska Native persons) are highly affected by substance use.^40,41^ In addition, our sample excluded patients with limited English proficiency and patients without addresses or telephones; these patients may have different SUD prevalence and diagnosis rates than those who enrolled in our study. Similarly, our sample represents VHA patients but may be less well-representative of other populations. However, although veterans are often considered at higher risk for SUDs than other populations, the prevalence of SUDs among veterans and subgroups of veterans is understudied. To our knowledge, the study findings represent the first population-based estimates of these diagnosis rates within key subgroups of veterans identified using a validated, structured assessment.

## Conclusions

A comparison of clinically documented diagnosis rates with survey-based prevalence rates across subgroups found substantial underrecognition of SUDs, particularly among women, younger persons, White non-Hispanic persons, and some non-White groups (eg, Hispanic and Latinx patients). Variations in diagnosis of SUDs may be associated with inequality in SUD care that cannot be identified and remedied using quality metrics specified with diagnosis-based denominators (eg, Healthcare Effectiveness Data and Evaluation Set measures).^42^ Further research should assess how patterns of underdiagnosis differ across contexts, and further research should explore these patterns of underdiagnosis and their underlying mechanisms within more-refined subgroups. Quality metrics for SUD treatment should be carefully chosen or redesigned to mitigate possible performance artifacts due to inequities in diagnosis that could stem from structural injustice.

## Notes

Supplement. **eTable 1.** Difference Between Clinically Documented Diagnoses and Survey-Based Prevalence of Alcohol Use Disorders (AUD), Drug Use Disorders (DUD), and Substance Use Disorders (SUD) in a Survey-Respondent VA Outpatient Population From 30 Geographically Diverse Facilities **eTable 2.** Clinically Documented Diagnosis Rates and Survey-Based Prevalence of Moderate/Severe Alcohol Use Disorders (AUD), Drug Use Disorders (DUD), and Substance Use Disorders (SUD) in a Survey-Respondent VA Outpatient Population From 30 Geographically Diverse Facilities
