# Supplementary material for: Comparison of Substance Use Disorder Diagnosis Rates From Electronic Health Record Data With Substance Use Disorder Prevalence Rates Reported in Surveys Across Sociodemographic Groups in the Veterans Health Administration
Source: JAMA Netw Open. 2022 Jun 30;5(6):e2219651. doi: 10.1001/jamanetworkopen.2022.19651 (PMC9247731; doi:10.1001/jamanetworkopen.2022.19651)
Supplement: Supplement. — eTable 1. Difference Between Clinically Documented Diagnoses and Survey-Based Prevalence of Alcohol Use Disorders (AUD), Drug Use Disorders (DUD), and Substance Use Disorders (SUD) in a Survey-Respondent VA Outpatient Population From 30 Geographically Diverse Facilities eTable 2. Clinically Documented Diagnosis Rates and Survey-Based Prevalence of Moderate/Severe Alcohol Use Disorders (AUD), Drug Use Disorders (DUD), and Substance Use Disorders (SUD) in a Survey-Respondent VA Outpatient Population From 30 Geographically Diverse Facilities [file jamanetwopen-e2219651-s001.pdf]

Supplementary Online Content

Williams EC, Fletcher OV, Frost MC, Harris AHS, Washington DL, Hoggatt KJ. Comparison of substance use disorder diagnosis rates from electronic health record data with substance use disorder prevalence rates reported in surveys across sociodemographic groups in the Veterans Health Administration. *JAMA Netw Open.* 2022;5(6):e2219651. doi:10.1001/jamanetworkopen.2022.19651

eTable 1. Difference Between Clinically Documented Diagnoses and Survey-Based Prevalence of Alcohol Use Disorders (AUD), Drug Use Disorders (DUD), and Substance Use Disorders (SUD) in a Survey-Respondent VA Outpatient Population From 30 Geographically Diverse Facilities
eTable 2. Clinically Documented Diagnosis Rates and Survey-Based Prevalence of Moderate/Severe Alcohol Use Disorders (AUD), Drug Use Disorders (DUD), and Substance Use Disorders (SUD) in a Survey-Respondent VA Outpatient Population From 30 Geographically Diverse Facilities

This supplementary material has been provided by the authors to give readers additional information about their work.

© 2022 Williams EC et al. *JAMA Network Open.*

eTable 1. Difference Between Clinically Documented Diagnoses and Survey-Based Prevalence of Alcohol Use Disorders (AUD), Drug Use Disorders (DUD), and Substance Use Disorders (SUD) in a Survey-Respondent VA Outpatient Population From 30 Geographically Diverse Facilities

	**AUD Clinically-Documented Diagnoses (Dx)**			**AUD Survey-Based Prevalence (Referent Standard) (Pr)**			**Difference Dx-Pr**	**Difference in Difference between subgroups**	
	**N**	**% (95% CI)**		**N**	**% (95% CI)**		**Difference (95% CI)**		**p-value**
**Overall**	360	6.0 (5.3-6.7)		608	10.1 (9.2-11.1)		-4.2 (-4.9, -3.3)	--	--
**Gender**									
Male	346	6.3 (5.5-7.2)		568	10.4 (9.5-11.4)		-4.1 (-4.9, -3.3)	Ref	
Female	14	2.5 (1.6-3.9)		40	7.0 (5.3-9.1)		-4.4 (-6.6, -2.4)	-0.3	0.76
**Age**									
18-34	27	6.9 (4.8-9.9)		88	22.4 (17.3-28.5)		-15.4 (-19.4, -11.6)	Ref	
35-49	86	10.1 (8.6-11.8)		153	18.1 (15.6-20.9)		-8.0 (-10.7, -5.2)	7.5	<0.01
50-64	123	8.1 (6.4-10.2)		170	11.2 (9.6-13.1)		-3.1 (-4.8, -1.4)	12.2	<0.01
65-74	104	5.1 (4.1-6.4)		161	7.9 (6.6-9.5)		-2.8 (-3.9, -1.6)	12.6	<0.01
75+	20	1.7 (1.1-2.6)		36	3.0 (2.2-4.1)		-1.3 (-2.5, -0.2)	14.1	<0.01
**Race/ethnicity**									
Black (NH)	95	9.0 (7.7-10.4)		139	13.0 (10.9-15.6)		-4.1 (-6.4, -1.8)	-0.5	0.68
Hispanic/Latinx	31	7.6 (5.3-10.8)		72	17.7 (14.0-22.1)		-10.0 (-13.9, -6.3)	-6.5	<0.01
White (NH)	206	5.0 (4.1-5.9)		353	8.6 (7.4-9.8)		-3.6 (-4.5, -2.7)	Ref	
Multirace	21	8.8 (5.7-13.4)		23	9.6 (6.6-13.8)		-0.8 (-4.6, 3.4)	2.8	0.17
Other	7	4.0 (2.2-7.4)		21	11.9 (8.7-16.2)		-7.9 (-13.4, -2.5)	-4.3	0.13
	**DUD Clinically-Documented Diagnoses (Dx)**			**DUD Survey-Based Prevalence (Referent Standard) (Pr)**			**Difference Dx-Pr**	**Difference in Difference between subgroups**	
	**N**	**% (95% CI)**		**N**	**% (95% CI)**		**Difference (95% CI)**		**p-value**
**Overall**	275	4.5 (3.7-5.6)		282	4.7 (3.9-5.6)		-0.1 (-0.8, 0.4)	--	--
**Gender**									
Male	259	4.7 (3.8-5.7)		264	4.8 (4.0-5.8)		-0.1 (-0.8, 0.5)	Ref	
Female	16	2.9 (1.6-5.3)		18	3.3 (2.1-5.1)		-0.4 (-2.0, 1.2)	-0.3	0.75
**Age**									
18-34	21	5.4 (3.7-7.8)		40	10.1 (7.2-14.0)		-4.7 (-8.1, -1.4)	Ref	
35-49	53	6.2 (5.0-7.7)		64	7.6 (5.8-9.8)		-1.4 (-3.2, 0.5)	3.3	0.08
50-64	109	7.2 (5.4-9.4)		102	6.7 (5.2-8.7)		0.5 (-1.0, 1.9)	5.2	0.01
65-74	81	4.0 (2.9-5.5)		72	3.6 (2.7-4.6)		0.4 (-0.6, 1.4)	5.1	<0.01
75+	11	0.9 (0.5-1.7)		4	0.3 (0.1-1.1)		0.6 (0.0, 1.1)	5.3	<0.01
**Race/ethnicity**									
Black (NH)	79	7.5 (6.0-9.3)		79	7.4 (5.6-9.8)		0.0 (-1.8, 1.9)	0.0	0.99
Hispanic/Latinx	28	6.8 (3.3-13.5)		33	8.1 (6.0-10.8)		-1.3 (-4.2, 1.7)	-1.4	0.36
White (NH)	139	3.3 (2.8-4.0)		136	3.3 (2.7-4.1)		0.0 (-0.6, 0.7)	Ref	
Multirace	18	7.5 (5.1-10.9)		21	8.7 (5.7-13.1)		-1.1 (-4.8, 2.6)	-1.2	0.52
Other	11	6.0 (3.1-11.4)		13	6.9 (3.9-11.7)		-0.9 (-4.6, 2.7)	-0.9	0.63
	**SUD Clinically-Documented Diagnoses (Dx)**			**SUD Survey-Based Prevalence (Referent Standard) (Pr)**			**Difference Dx-Pr**	**Difference in Difference between subgroups**	

© 2022 Williams EC et al. *JAMA Network Open.*

	N	% (95% CI)		N	% (95% CI)		Difference (95% CI)		p-value
**Overall**	515	8.5 (7.5-9.6)		768	12.8 (11.7-13.9)		-4.2 (-5.1, -3.3)	--	--
**Gender**									
Male	490	8.9 (7.9-10.1)		716	13.1 (12.0-14.3)		-4.2 (-5.1, -3.2)	Ref	
Female	25	4.5 (3.0-6.6)		52	9.2 (7.2-11.8)		-4.7 (-7.3, -2.3)	-0.5	0.66
**Age**									
18-34	41	10.5 (8.1-13.4)		109	27.7 (22.6-33.3)		-17.2 (-21.7, -12.8)	Ref	
35-49	110	12.9 (11.2-14.8)		187	22.1 (19.6-24.8)		-9.2 (-12.0, -6.3)	8.0	<0.01
50-64	180	11.8 (9.7-14.3)		230	15.2 (13.5-17.1)		-3.3 (-5.2, -1.4)	13.9	<0.01
65-74	155	7.6 (6.2-9.3)		203	10.0 (8.5-11.7)		-2.4 (-3.8, -1.0)	14.8	<0.01
75+	29	2.4 (1.7-3.4)		39	3.2 (2.3-4.5)		-0.8 (-2.0, 0.3)	16.4	<0.01
**Race/ethnicity**									
Black (NH)	137	13.0 (11.2-15.0)		182	17.1 (14.8-19.6)		-4.1 (-6.6, -1.7)	-0.5	0.69
Hispanic/Latinx	48	11.7 (7.9-16.9)		88	21.6 (18.0-25.8)		-10.0 (-14.4, -5.8)	-6.4	<0.01
White (NH)	288	6.9 (6.1-7.9)		434	10.5 (9.4-11.8)		-3.6 (-4.6, -2.6)	Ref	
Multirace	29	12.2 (9.0-16.4)		36	15.0 (11.3-19.7)		-2.7 (-7.5, 1.6)	0.8	0.72
Other	13	7.3 (4.5-11.4)		28	15.6 (11.5-20.9)		-8.4 (-14.3, -2.7)	-4.7	0.10

© 2022 Williams EC et al. *JAMA Network Open.*

eTable 2. Clinically Documented Diagnosis Rates and Survey-Based Prevalence of Moderate/Severe Alcohol Use Disorders (AUD), Drug Use Disorders (DUD), and Substance Use Disorders (SUD) in a Survey-Respondent VA Outpatient Population From 30 Geographically Diverse Facilities

	**AUD Clinically-Documented Diagnoses (Dx)**			**Moderate to Severe AUD Survey-Based Prevalence (Referent Standard) (Pr)**			**Sensitivity**	**Absolute difference Sensitivity**	**Specificity**	**Absolute difference Specificity**
	**N**	**% (95% CI)**		**N**	**% (95% CI)**			**p-value**		**p-value**
**Overall**	360	6.0 (5.3-6.7)		311	5.1 (4.5-5.8)		42.5 (37.9-47.3)		96.0 (95.5-96.5)	
**Gender**										
Male	346	6.3 (5.5-7.2)		544	5.3 (4.6-6.0)		43.3 (38.3-48.5)	0.3199	95.8 (95.2-96.8)	<0.0001
Female	14	2.5 (1.6-3.9)		22	3.8 (2.7-5.5)		31.6 (14.4-55.9)		98.7 (97.3-99.3)	
**Age**										
18-34	27	6.9 (4.8-9.9)		53	13.5 (10.2-17.7)		33.8 (23.4-46.0)	0.1757	97.3 (94.8-98.6)	<0.0001
35-49	86	10.1 (8.6-11.8)		81	9.4 (7.5-11.8)		41.8 (31.4-53.0)		93.2 (91.4-94.7)	
50-64	123	8.1 (6.4-10.2)		94	6.2 (5.0-7.7)		43.5 (31.1-56.8)		94.2 (92.4-95.7)	
65-74	104	5.1 (4.1-6.4)		70	3.5 (2.6-4.6)		50.9 (39.3-62.4)		96.5 (95.7-97.2)	
75+	20	1.7 (1.1-2.6)		13	1.1 (0.6-2.0)		30.7 (12.0-58.9)		98.6 (97.8-99.1)	
**Race/ethnicity**										
Black (NH)	95	9.0 (7.7-10.4)		73	6.9 (5.4-8.6)		50.9 (42.3-59.4)	0.0036	94.1 (92.3-95.6)	0.0136
Hispanic/Latinx	31	7.6 (5.3-10.8)		43	10.5 (7.0-15.5)		27.4 (16.3-42.2)		94.7 (92.0-96.6)	
White (NH)	206	5.0 (4.1-5.9)		177	4.3 (3.5-5.2)		43.5 (38.6-48.5)		96.8 (96.2-97.3)	
Multirace	21	8.8 (5.7-13.4)		10	4.1 (2.6-6.6)		58.5 (26.8-84.4)		93.3 (89.2-95.9)	
Other	7	4.0 (2.2-7.4)		8	4.3 (2.6-7.2		11.6 (1.3-56.3)		96.3 (93.0-98.1)	
	**DUD Clinically-Documented Diagnoses (Dx)**			**Moderate to Severe DUD Survey-Based Prevalence (Referent Standard) (Pr)**			**Sensitivity**	**Absolute difference Sensitivity**	**Specificity**	**Absolute difference Specificity**
	**N**	**% (95% CI)**		**N**	**% (95% CI)**			**p-value**		**p-value**
**Overall**	275	4.5 (3.7-5.6)		132	2.2 (1.8-2.7)		48.4 (36.9-60.1)		96.4 (95.7-97.1)	
**Gender**										
Male	259	4.7 (3.8-5.7)		126	2.3 (1.8-2.8)		48.3 (35.5-61.3)	0.9157	96.3 (95.6-97.0)	0.0479
Female	16	2.9 (1.6-5.3)		6	1.2 (1.8-2.7)		50.4 (22.3-77.8)		97.7 (95.5-98.8)	
**Age**										
18-34	21	5.4 (3.7-7.8)		13	3.4 (1.9-6.0)		31.2 (11.4-61.5)	0.5474	95.5 (93.5-96.9)	<0.0001
35-49	53	6.2 (5.0-7.7)		43	5.1 (3.8-6.9)		50.5 (35.5-65.5)		96.2 (95.1-97.1)	
50-64	109	7.2 (5.4-9.4)		44	2.9 (2.0-4.2)		45.5 (27.8-64.5)		94.0 (92.1-95.4)	
65-74	81	4.0 (2.9-5.5)		30	1.5 (1.1-2.0)		57.0 (42.0-70.9)		96.8 (95.4-97.8)	
75+	11	0.9 (0.5-1.7)		2	0.2 (0.0-0.7)		50.0 (5.0-95.0)		99.2 (98.4-99.6)	

© 2022 Williams EC et al. *JAMA Network Open.*

	**DUD Clinically-Documented Diagnoses (Dx)**			**Moderate to Severe DUD Survey-Based Prevalence (Referent Standard) (Pr)**			**Sensitivity**	**Absolute difference Sensitivity**	**Specificity**	**Absolute difference Specificity**
	**N**	**% (95% CI)**		**N**	**% (95% CI)**			**p-value**		**p-value**
**Race/ethnicity**										
Black (NH)	79	7.5 (6.0-9.3)		45	4.3 (3.2-5.7)		50.9 (37.4-64.2)	0.5408	94.5 (92.8-95.8)	0.0075
Hispanic/Latinx	28	6.8 (3.3-13.5)		15	3.7 (2.5-5.4)		59.6 (27.2-85.4)		95.3 (90.0-97.8)	
White (NH)	139	3.3 (2.8-4.0)		58	1.4 (1.0-1.9)		39.5 (25.3-55.7)		97.2 (96.5-97.7)	
Multirace	18	7.5 (5.1-10.9)		6	2.6 (1.2-5.6)		67.6 (23.2-93.5)		94.1 (91.0-96.2)	
Other	11	6.0 (3.1-11.4)		8	4.5 (2.1-9.4)		61.3 (25.5-88.0)		96.6 (91.2-98.7)	
	**SUD Clinically-Documented Diagnoses (Dx)**			**Moderate to Severe SUD Survey-Based Prevalence (Referent Standard) (Pr)**			**Sensitivity**	**Absolute difference Sensitivity**	**Specificity**	**Absolute difference Specificity**
	**N**	**% (95% CI)**		**N**	**% (95% CI)**			**p-value**		**p-value**
**Overall**	515	8.5 (7.5-9.6)		390	6.5 (5.7-7.3)		46.8 (41.7-52.0)		94.1 (93.3-94.9)	
**Gender**										
Male	490	8.9 (7.9-10.1)		364	6.6 (5.8-7.5)		47.9 (42.1-53.7)	0.1338	93.9 (93.0-94.6)	0.0021
Female	25	4.5 (3.0-6.6)		26	4.7 (3.5-6.3)		31.1 (15.7-52.3)		96.7 (94.6-98.2)	
**Age**										
18-34	41	10.5 (8.1-13.4)		61	15.6 (11.8-20.3)		34.4 (24.4-46.1)	0.0315	94.0 (91.2-95.9)	<0.0001
35-49	110	12.9 (11.2-14.8)		106	12.4 (10.2-15.1)		45.0 (35.5-54.9)		91.2 (89.5-93.4)	
50-64	180	11.8 (9.7-14.3)		118	7.8 (6.5-9.3)		48.5 (35.9-61.2)		91.3 (88.9-93.1)	
65-74	155	7.6 (6.2-9.3)		91	4.5 (3.5-5.7)		56.7 (47.3-65.7)		94.7 (93.4-95.7)	
75+	29	2.4 (1.7-3.4)		14	1.2 (0.6-2.1)		35.6 (14.2-65.0)		98.0 (97.0-98.6)	
**Race/ethnicity**										
Black (NH)	137	13.0 (11.2-15.0)		100	9.4 (8.1-10.9)		55.1 (46.7-63.4)	0.0181	91.4 (89.2-93.2)	0.0109
Hispanic/Latinx	48	11.7 (7.9-16.9)		49	12.0 (8.3-17.1)		36.3 (23.6-51.2)		91.7 (87.2-94.7)	
White (NH)	288	6.9 (6.1-7.9)		213	5.1 (4.3-6.2)		44.1 (39.8-49.5)		95.1 (94.3-95.7)	
Multirace	29	12.2 (9.0-16.4)		15	6.3 (4.2-9.4)		67.1 (43.3-84.5)		91.5 (87.6-94.3)	
Other	13	7.3 (4.5-11.4)		13	7.2 (4.5-11.4)		44.7 (24.5-66.8)		95.7 (91.3-97.9)	

© 2022 Williams EC et al. *JAMA Network Open.*
